# Corneal crosslinking via the Dresden protocol versus the accelerated approach in pediatric patients - a retrospective comparative study

**DOI:** 10.5935/0004-2749.2023-0309

**Published:** 2024-10-31

**Authors:** Júlia Maggi Vieira, Isadora Brito Coelho, Fellype Borges de Oliveira, Heloisa Nascimento, Fábio Nishimura Kanadani, Evandro Ribeiro Diniz

**Affiliations:** 1 Glaucoma Instituto, Belo Horizonte, MG, Brazil; 2 Setor de Córnea, Instituto de Olhos Ciências Médicas, Belo Horizonte, MG, Brazil; 3 Setor de Córnea e Segmento Anterior, Banco de Olhos de Sorocaba, Sorocaba, SP, Brazil; 4 Setor de Córnea, Universidade Federal de São Paulo, São Paulo, SP, Brazil; 5 Faculdade de Medicina, Universidade de São Paulo, São Paulo, SP, Brazil

**Keywords:** Keratoconus, Corneal diseases, Ultraviolet rays, Cross-linking reagents, Visual acuity

## Abstract

**Purpose:**

Keratoconus presents certain peculiarities in pediatric patients when compared with adults. The greatest challenge in children is that the disease is more severe and faster in progression. In this retrospective study, we aimed to compare the accelerated and Dresden protocols for corneal crosslinking in patients aged <18 years who were followed-up for at least 12 months. **Method**s: A total of 36 eyes from 27 patients were included in the study. The best corrected and uncorrected visual acuity, maximal keratometry, corneal thickness, foveal thickness, and endothelial microscopy findings were evaluated at baseline and during the postoperative period at one, three, and six months. Thereafter, the patients were evaluated at one, three, six and twelve months postoperative. Corneal crosslinking was performed in all patients via the Dresden protocol (n=21 eyes) or the accelerated protocol (n=15 eyes). Data between the two groups were compared and XY statistical analysis was used.

**Results:**

Both protocols were effective in halting keratoconus progression. No patient had progression at the 12-month follow-up. A significant reduction in Kmax and improvement in the corrected distance visual acuity were observed only in the Dresden protocol group. Although the Dresden protocol was superior to the accelerated protocol in reducing Kmax (p=0.002), there was no significant difference in corrected distance visual acuity between the two groups.

**Conclusion:**

The accelerated protocol is as efficient as the Dresden protocol in stabilizing keratoconus progression. Although the Dresden protocol was superior to the accelerated protocol in reducing the Kmax, it did not produce better clinical results. Thus, the accelerated protocol is an efficient option. Furthermore, considering the advantages of reduced surgical time, the accelerated protocol is effective in halting keratoconus progression in the pediatric age group.

## INTRODUCTION

Keratoconus (KC) is a noninflammatory, chronic, progressive, bilateral and often asymmetrical disease wherein the cornea becomes conical in shape due to the biomechanical instability of corneal collagen fibers^(1^,^2)^. The condition affects all ethnicities and sexes. Although it is commonly an isolated ocular condition, it sometimes coexists with other ocular and systemic diseases such as atopy^(2)^.

The classic histopathologic features of KC include stromal thinning, iron deposition in the epithelial basement membrane, and breaks in the Bowman’s layer. As the disease advances, the patient presents with variable visual impairment, irregular astigmatism, or myopia. KC mostly appears during puberty and gradually progresses until the fourth decade of life. Furthermore, KC is usua-lly more advanced at the time of diagnosis and more aggressive in progression in children than in adults. This may be attributable to the eye rubbing and eye allergy, which are more common in children than in adults^(3^-^6)^.

KC significantly impacts the patients’ quality of life. It is even more significant in children due to the impact that visual impairment may have on social and educational development. KC can be treated with glasses, contact lenses, corneal crosslinking (CXL), and keratoplasty. However, fitting rigid lenses in children can be challenging. Furthermore, corneal transplantation should be postponed as much as possible. Therefore, preventing the progression of KC in the early stages is essential^(6)^.

Corneal CXL has been used to halt the progression of KC since the 2000s in an attempt to avoid lamellar or penetrating keratoplasty^(5^,^7)^. The treatment is based on the irradiation of riboflavin that has been activated by ultraviolet-A (UV-A) light to strengthen the corneal stroma collagen fibers^(3^,^8^-^11)^. The standard protocol, also known as the Dresden protocol, involves irradiation with UV-A light for 30 minutes at 3.0 mW/cm^2^ to deliver a fluence of 5.4 J/cm^2^. Corneal CXL is widely used, effective, and secure^(12^,^13)^. However, accelerated protocols that decrease the time of corneal exposure and potential intraoperative risks have been recently studied. Corneal haze, corneal scars, mild photophobia, and blepharitis are some of the possible complications of CXL. The same energy (5.4 J/cm^2^) and faster effect can also be achieved with a higher UVA irradiation intensity^(1)^. There are several studies on this promising new approach. However, studies comparing both protocols in the pediatric population are limited^(14^,^15)^. Thus, in this study, we aimed to compare the efficacy and safety of accelerated CXL with those of the conventional method in patients aged <18 years.

## METHODS

In this study, we retrospectively reviewed the charts of 36 eyes from 27 patients, aged <18 years, who had undergone corneal collagen CXL for progressive KC at a private ophthalmic clinic in Belo Horizonte, Minas Gerais, Brazil, between January 2017 and January 2020. This study was approved by the Research Ethics Committees of *Faculdade Ciências Médicas de Minas Gerais* (No: 40344020.6.0000.5134; 5 April 2021) and *Hospital Universitário Ciências Médicas, MG, Brazil* (No: 90113418.0.0000.5134).

All patients had been diagnosed with KC on the basis of clinical findings, biomicroscopic signs, and Rabinowitz criteria, which is based on the placid anterior curvature of the cornea^(2)^. The diagnosis was confirmed via corneal tomography (Pentacam^®^, OCULUS, Wetzlar, Germany) during the subsequent follow-ups.

The following were the inclusion criteria: progressive KC (an increase of ≥0.75 D over 6 months or >1.0 D over 1 year in the corneal apical topographical keratometry), minimum central corneal thickness (CCT) of 400 µm, absence of other anterior segment diseases and other systemic pathologies that could interfere with corneal healing, absence of previous surgeries or procedures in the eye, attendance at all biannual appointments, and a postoperative period of at least 1 year.

The following preoperative and postoperative data were also collected: best corrected and uncorrected visual acuity (logMAR), corneal thickness, and maximum keratometry (Kmax: the most curved part of the cornea) (Table 1).The differences between the 6 months and pre and 12 months preoperative and pre periods were obtained for each studied variable 6- and 12-month postoperative Kmax, CDVA, and corneal thickness values in the two protocol groups were obtained and these differences were compared.

**Table 1:** Preoperative and postoperative visual acuity and corneal assessments in the two interventions groups

Protocol	Variable	Preoperative	6 months postoperative	12 months postoperative	p-value	
					Pre x 6 month	Pre x 12 month
Dresden	Kmax	54.9 (52.1-61.4)	53.8 (51.3-60.9)	52.8 (50.4-60.5)	**0.003**	**0.004**
	CDVA (LogMAR)	0.20 (0.15-0.35)	0.20 (0.10-0.20)	0.10 (0.10-0.20)	0.059	**0.010**
	Corneal thickness (Pachymetry)	441 (429-469)	421 (412-433)	424 (413-439)	**<0.001**	**0.003**
Accelerated	Kmax	52.0 (50.8-60.4)	56.9 (51.8-62.2)	55.0 (51.1-60.3)	0.363	0.169
	CDVA (LogMAR)	0.30 (0.10-0.50)	0.20 (0.18-0.42)	0.20 (0.20-0.30)	0.272	0.603
	Corneal thickness (Pachymetry)	444 (429-473)	444 (427-466)	451 (435-468)	0.861	0.838

Data as presented as median (1st-3rd quartiles).

Kmax= maximum keratometry; CDVA= corrected distance visual acuity.

Patients using contact lenses were advised to suspend their use at least 2 weeks before the examination. Patients with an intrastromal ring implant and incomplete follow-up were excluded from the study.

CXL was performed under sterile conditions by the same surgeon (ERD) according to the Dresden protocol. Topical anesthesia (proxymetacaine) was instilled 5 min before the procedure and immediately before the procedure (1 drop each). Thereafter, 8-10 µm of the corneal epithelium was removed. Subsequently, riboflavin (0.1% riboflavin-5-phosphate and 20% dextran T-500) was applied 30 minutes before irradiation and every 5 minutes during irradiation. No general anesthesia or sedation was performed. The accelerated CXL was performed in a similar way. However, 0.1% riboflavin-5-phosphate with hydroxypropylmethylcellulose (HPMC) was applied for 16 minutes, followed by UVA irradiation for 10 minutes at an intensity of 9 mW/cm^2^. An extra drop of riboflavin was instilled 5 minutes after the Dresden Protocol and 2 minutes after the accelerated protocol.

During the postoperative period, a topical antibiotic (moxifloxacin) and steroid (dexamethasone) were instilled four times a day for 10 days. Additionally, the patients were advised to wear therapeutic contact lenses for 5 days. All the patients and their parents or guardians were informed of the risks and benefits of the procedure, and informed consent was obtained from them. The study was conducted in accordance with the tenets of the Declaration of Helsinki.

### Statistics

The numerical variables that exhibited normal Gaussian distribution are expressed as means and standard deviations. The variables that did not exhibit normal Gaussian distribution are expressed as medians and first and third quartiles. The categorical variables are expressed as frequencies and percentages. The assumption of normal distribution was verified using the Shapiro-Wilk test.

The nonparametric Wilcoxon test was used to compare numerical variables between the same groups at different times. The nonparametric Mann-Whitney test was used to compare numerical variables between diffe-rent groups. All statistical analyses were performed using SPSS (version 23). Statistical significance was set at p<0.05.

## RESULTS

Thirty-six eyes from 27 patients were included in the study. Of the 36 eyes, 21 (58.3%) belonged to females and 15 (41.7%) belonged to males. Furthermore, 21 (58.3%) eyes were included in the Dresden protocol, and 15 (41.7%) eyes in the accelerated protocol. The mean age of the patients in the Dresden protocol and accelerated protocol was 14.62 ± 1.96 years and 14.33 ± 2.90 years, respectively. There were no significant diffe-rences in the demographic variables between the two groups (p>0.05; Table 2). Table 1 includes the medians and quartiles of each analyzed variable (Kmax, corrected distance visual acuity [CDVA], and CCT) from the preoperative period to the 1-year follow-up.

**Table 2:** Demographic characteristics of the patients who underwent CXL via the Dresden and accelerated protocols

Variables	Total (n=36)	Dresden group (n=21)	Accelerated group (n=15)	p-value
Age	**14.50 ± 2.36**	**14.62 ± 1.96**	**14.33 ± 2.90)**	**0.726**
Sex	**36**	**21**	**15**	**0.391**
Female	15 (41.7)	10 (47.6)	5 (33.3)	
Male	21 (58.3)	11 (52.4)	10 (66.7)	
Eye	**36**	**21**	**15**	**0.955**
Right	19 (52.8)	11 (52.4)	8 (53.3)	
Left	17 (47.2)	10 (47.6)	7 (46.7)	

Data are presented as frequency (%) or mean ± mean deviation.

**Figure 1:**
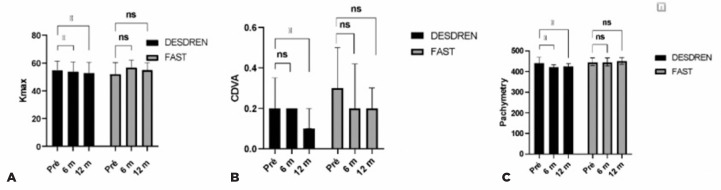
Comparison of the preoperative and 6- and 12-month postoperative (A) Kmax, (B) CDVA and (C) corneal thickness values in the two intervention groups.

In the Dresden protocol, the Kmax had significantly reduced from the initial value (median, 54.9) by the 6-month (median, 53.9; p=0.003) and 1-year (median, 52.8; p=0.004) follow-up (Figure 1A). At the 12^th^ month, the CDVA had improved from a median of 0.20 to 0.10, corresponding to an approximate gain of one line of vision. This increase was statistically significant (p=0.010). However, the improvement at the 6th month was not significantly different from the baseline (p=0.059; Figure 1B). Additionally, there was also a statistically significant reduction in the corneal thickness at the thinnest point, both at the 6-month (reduction from 441 to 421 µm; p<0.001) and 1-year (reduction from 441 to 424 µm; p=0.003) follow-ups (Figure 1C).

In the accelerated protocol, there was no statistically significant difference in the Kmax, CDVA, or corneal thickness at the 6-month and 1-year follow-up (Figure 1).

**Figure 2:**
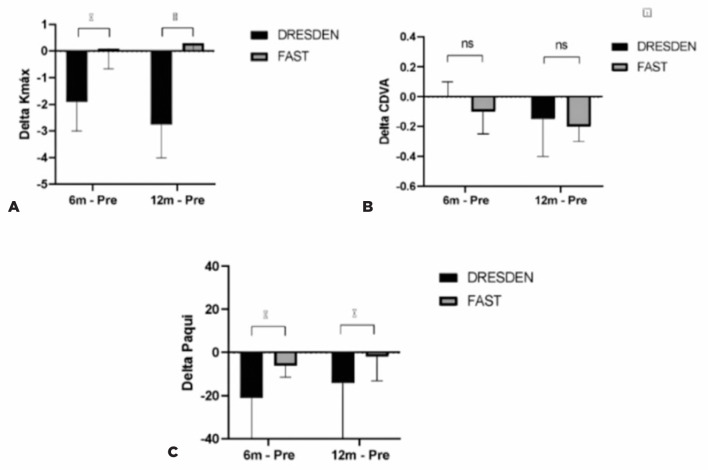
Mean delta values of the (A) Kmax, (B) CDVA, and (C) corneal thickness at the 6-month and 12-month follow-up in the Dresden and accelerated protocols.

The Kmax and CCT were significantly different between the two protocols at the 6-month and 12-month follow-up (Figures 2A-B). However, the CDVA was not significantly different between the two protocols (Figure 2C).

There was a greater median reduction in the Kmax in the Dresden group than in the accelerated protocol group at the 6-month (delta, -1.9 x 0.10; p=0.002) and 12-month (delta, -2.75 x 0.3; p=0.001) follow-ups (Table 3). Additionally, there was a greater median reduction in the CCT in the Dresden group than in the accelerated protocol group at the 6-month (delta, -21.0 x -6.0; p=0.002) and 12-month (delta, -14.0 x -2.0; p=0.019) follow-ups. However, the difference in the CDVA was not statistically significant between the two groups at the 6-month (delta, 0.00 x -0.10; p=0.635) and 12-month (-0.15 x -0.20; p=0.868) follow-ups.

**Table 3:** Comparison of the delta values in the Dresden and accelerated protocol groups

Variables	Dresden	Accelerated	p-value
Delta Kmax (6m-Pre)	-1.9 (-3.0; -0.35)	0.10 (-0.65; 1.33)	0.002^*^
Delta Kmax (12m-Pre)	-2.75 (-4.0;-0.63)	0.3 (0.0; 2.30)	0.001^*^
Delta CDVA (6m-Pre)	0.00 (-0.35; 0.10)	-0.10 (-0.25; 0.03)	0.635
Delta CDVA (12m-Pre)	-0.15 (-0.40; 0.10)	-0.20 (-0.30; 0.00)	0.868
Delta Pachy (6m-Pre)	-21.0 (-46.0; -7.0)	-6.0 (-11.3;19.8)	0.002^*^
Delta Pachy (12m-Pre)	-14.0 (-43.3; -4.25)	-2.0 (-13.0; 12.0)	0.019^*^

6m= 6-month value; Pre= preoperative value; 12m= 12-month values; (Kmax= maximum keratometry; CDVA= corrected distance visual acuity; Pachy= pachymetry.

## DISCUSSION

KC is a degenerative corneal disorder characterized by irregular astigmatism, corneal thinning, and visual impairment. It tends to be more severe and progressive in pediatric patients than in adults^(1^,^2)^. Generally, the disease begins in the second decade of life, and it may progress until the fourth decade of life^(16)^.

The main risk factors for the rapid progression of KC are an age of <17 years and Kmax >55 D at the time of diagnosis^(17)^. In general, children tend to have more ocular allergies and poorer pruritus control, which are directly involved in the pathophysiology of the disease, than adults. Furthermore, the progression of KC tends to decrease with age due to the natural crosslinking of corneal collagen, which increases corneal rigidity^(18)^.

Considering the fact that the younger population is at greater risk, it is crucial to study the effects of corneal CXL and determine the ideal treatment^(19)^. CXL is effective in halting the progression of KC. However, studies have reported divergent results regarding the efficacy of CXL in adults and children^(6^,^20)^. Despite the evidence of its effectiveness, there is still no consensus on when CXL is indicated.

In this study, a criterion for CXL progression was an increase in Kmax of ≥0.75 D over 6 months or >1.0 D over 1 year^(21)^. Moreover, obtaining reliable and reproducible information on visual acuity and imaging tests to determine disease progression becomes more challenging as the age decreases. CXL has some complications. Thus, it should be recommended only to patients who show signs of progression. However, it is impossible to predict which patients will develop disease progression.

There are different ways to perform CXL such as removing or retaining the epithelium, using riboflavin with different vehicles, and irradiating with different fluences and durations^(22)^. In the Dresden protocol, the vehicle used is 0.1% riboflavin-5-phosphate in 20% dextran T-500. In the accelerated protocol, the vehicle used is 0.1% riboflavin-5-phosphate in HPMC^(23^,^24)^. These are the commonly used protocols currently, and there are studies showing the effectiveness of both^(25^,^26)^. Because the procedure requires patient cooperation, the accelerated protocol tends to be more advantageous than the Dresden protocol, especially in children. Thus, comparing the two protocols in this age group is important to determine the appropriate indications^(25^,^26)^.

In a previous study, the effectiveness of the Dresden protocol in patients aged <18 years was assessed. The study results were consistent with those of other studies, indicating a trend toward a reduction in Kmax and improvement in CDVA over the 24-month postoperative period. The current study findings also reveal a trend of improvement in CDVA and a statistically significant reduction in corneal thickness^(4)^.

A 2018 meta-analysis study that analyzed 1,158 eyes did not find a significant difference in the Kmax and CDVA between the Dresden and accelerated protocols. However, in this study, corneal thinning was lesser in the accelerated protocol than in the Dresden protocol. However, the meta-analysis identified a deeper demarcation line and greater effect on the minimum keratometry in the conventional protocol than in the acceleration protocol, indicating that the conventional protocol was more effective^(23)^. Another meta-analysis by Huang et al., which included 713 eyes, showed a greater reduction in Kmax in the conventional protocol and a smaller reduction in corneal thickness and endothelial count in the accelerated protocol^(24)^. However, these studies do not stratify the results by age group. Thus, considering the particularities of KC progression in children, it cannot be concluded that the results in each age group would be the same.

A 2016 meta-analysis study demonstrated that the Kmax stabilized and visual acuity improved in the conventional protocol (seven studies) with and without correction at the 1-year follow-up. In this same meta--analysis, two studies in which the accelerated protocol was assessed were included. In one study, 18 eyes were subjected to UVA light at 9 mW/cm^2^ for 10 minutes. In the other study, 61 eyes were subjected to UVA light at 30 mW/cm^2^ for 4 minutes. In both these studies, there was a significant improvement in the uncorrected visual activity, best corrected visual activity, and Kmax at the 2-year follow-up^(27)^.

There have been only a few studies till date that have compared the results of CXL using the conventional and accelerated protocols in the pediatric age group. This evaluation is crucial because the effectiveness of the protocols may differ with age groups because KC progression is more intense among children and adolescents than among adults. In this study, both protocols were effective in fulfilling the primary function of CXL, which is to stabilize the progression of KC.

The study results also demonstrated a statistically significant reduction in Kmax of 1 D and 2 D at the 6-month and 12-month follow-up, respectively, in the Dresden protocol group. Moreover, the CDVA had improved from 20/30 to 20/25 at the 1-year follow-up, corresponding to a gain of 1 line of vision. In the accelerated protocol group, there were no significant changes in Kmax or CDVA, indicating an absence of KC progression. However, the primary objective of CXL is the stabilization of the disease, which can be achieved via either protocol. Although corneal flattening is, in principle, a desirable secondary outcome, extreme progressive flattening is associated with greater corneal thinning, especially in patients with a higher preoperative Kmax^(28)^.

In the Dresden protocol group, the thinnest corneal point exhibited a reduction of 20 microns after 6 months. No further thinning occurred between the 6th month and the 1-year follow-up. However, there was no significant change in corneal thickness in the accelerated group. Additionally, the consequences of this corneal thinning, if clinically relevant, remain unknown. Although we observed a significant improvement in CDVA in the Dresden protocol group after 6 and 12 months, this improvement was not statistically superior to the change in the accelerated protocol group.

Long-term follow-up is essential to validate our results in children. Despite the noticeable improvement in parameters after CXL, the present study was limited by its retrospective design, the short follow-up duration, and no recurrence of progression or future complications.

Several studies have demonstrated that the disease can progress even after CXL, mainly due to persistent rubbing of the eyes and/or spring keratoconjunctivitis^(14)^. Furthermore, risks such as corneal scarring, abrasion--related discomfort, blepharitis, and photophobia cannot be excluded, despite previous studies demonstrating that CXL in children is as safe as CXL in adolescents and adults^(29)^. Nevertheless, further studies are required to determine whether CXL is as effective in the pediatric population as it is in adults in the long run. Furthermore, the possibility of retreatment needs to be analyzed. In addition, the use of higher energy than that used in the standard protocol to increase the treatment efficacy needs to be evaluated.

In conclusion, KC in children typically evolves rapidly with significant visual impairment. Therefore, the control that corneal CXL provides over ectasia progression becomes paramount in this age group, which could reduce the need for penetrating keratoplasty. Nevertheless, the significance of early diagnosis and short-term follow-up (every 3 months, according to most authors) need to be highlighted.

Both the Dresden and accelerated protocols are effective in stabilizing the progression of KC. The Dresden protocol has the advantage of being around longer in clinical practice. Therefore, its results in the literature are more robust with longer follow-up periods. The accelerated protocol offers faster execution, which is advantageous for physicians seeking to optimize their time and improve patient comfort. This is especially relevant in the pediatric population because patient cooperation is essential for proper treatment.

In this study, a greater reduction in Kmax was observed in the Dresden protocol group than in the accelerated protocol group. Thus, the Dresden protocol may be superior to the accelerated protocol in terms of corneal crosslinking. Furthermore, the BCVA had improved at the 12-month follow-up in the Dresden protocol group. However, this was not statistically significantly different from the results of the accelerated protocol group. Considering the fact that the primary objective of CXL is to stop KC progression, neither protocol is superior to the other in terms of effectiveness. However, considering the advantage of reduced surgical time, the accelerated protocol can be an effective option to halt KC progression in the pediatric age group.

## References

[1] Medeiros CS, Giacomin NT, Bueno RL, Ghanem RC, Moraes HV (2016). Santhiago MR. Accelerated corneal collagen crosslinking:
Technique, efficacy, safety, and applications. J Cataract Refract Surg.

[2] Rabinowitz YS. (1998). Keratoconus. Surv Ophthalmol.

[3] McAnena L, O’Keefe M. (2015). Corneal collagen crosslinking in children with
keratoconus. J AAPOS.

[4] Diniz ER, Barbosa JC, Jacometti R, Souza RT, Kanadani FN. (2021). Corneal crosslinking efficacy in patients with keratoconus under
18 years of age. Arq Bras Oftalmol.

[5] Raiskup F, Theuring A, Pillunat LE, Spoerl E. (2015). Corneal collagen crosslinking with riboflavin and ultraviolet-A
light in progressive keratoconus: ten-year results. J Cataract Refract Surg.

[6] Mukhtar S, Ambati BK. (2018). Pediatric keratoconus: a review of the literature. Int Ophthalmol.

[7] Gomes JA, Tan D, Rapuano CJ, Belin MW, Ambrósio R, Guell JL, Group of Panelists for the Global Delphi Panel of Keratoconus and
Ectatic Diseases (2015). Global consensus on keratoconus and ectatic
diseases. Cornea.

[8] Zotta PG, Moschou KA, Diakonis VF, Kymionis GD, Almaliotis DD, Karamitsos AP (2012). Corneal collagen cross-linking for progressive keratoconus in
pediatric patients: a feasibility study. J Refract Surg.

[9] Caporossi A, Mazzotta C, Baiocchi S, Caporossi T, Denaro R, Balestrazzi A. (2012). Riboflavin-UVA-induced corneal collagen cross-linking in
pediatric patients. Cornea.

[10] Chatzis N, Hafezi F. (2012). Progression of keratoconus and efficacy of corneal collagen
cross-linking in children and adolescents. J Refract Surg.

[11] Mazzotta C, Traversi C, Baiocchi S, Bagaglia S, Caporossi O, Villano A (2018). Corneal collagen cross-linking with riboflavin and ultraviolet A
light for pediatric keratoconus: ten-year results. Cornea.

[12] Peyman A, Kamali A, Khushabi M, Nasrollahi K, Kargar N, Taghaodi M (2015). Collagen cross-linking effect on progressive keratoconus in
patients younger than 18 years of age: A clinical trial. Adv Biomed Res.

[13] Wise S, Diaz C, Termote K, Dubord PJ, McCarthy M, Yeung SN. (2016). Corneal cross-linking in pediatric patients with progressive
keratoconus. Cornea.

[14] Shah R, Shah S, Sengupta S. (2011). Results of small incision lenticule extraction: all-in-one
femtosecond laser refractive surgery. J Cataract Refract Surg.

[15] Pallikaris IG, Portaliou DM, Kymionis GD, Panagopoulou SI, Kounis GA. (2014). Outcomes after accommodative bioanalogic intraocular lens
implantation. J Refract Surg.

[16] Gomes BA, Santhiago MR, Jorge PA, Kara-José N, Moraes HV (2015). Kara-Junior N. Corneal involvement in systemic inflammatory
diseases. Eye Contact Lens.

[17] Ferdi AC, Nguyen V, Gore DM, Allan BD, Rozema JJ, Watson SL. (2019). Keratoconus natural progression: a systematic review and
meta--analysis of 11 529 eyes. Ophthalmology.

[18] El Rami H, Chelala E, Dirani A, Fadlallah A, Fakhoury H, Cherfan C (2015). An Update on the safety and efficacy of corneal collagen
cross-linking in pediatric keratoconus. BioMed Res Int.

[19] Krachmer JH, Feder RS, Belin MW. (1984). Keratoconus and related noninflammatory corneal thinning
disorders. Surv Ophthalmol.

[20] Peyman A, Kamali A, Khushabi M, Nasrollahi K, Kargar N, Taghaodi M (2015). Collagen cross-linking effect on progressive keratoconus in
patients younger than 18 years of age: A clinical trial. Adv Biomed Res.

[21] Kankariya VP, Kymionis GD, Diakonis VF, Yoo SH. (2013). Management of pediatric keratoconus - evolving role of corneal
collagen cross--linking: an update. Indian J Ophthalmol.

[22] Montana CL, Bhorade AM. (2018). Glaucoma and quality of life: fall and driving
risk. Curr Opin Ophthalmol.

[23] Shajari M, Kolb CM, Agha B, Steinwender G, Müller M, Herrmann E (2019). Comparison of standard and accelerated corneal cross--linking for
the treatment of keratoconus: a meta-analysis. Acta Ophthalmol.

[24] Wen D, Li Q, Song B, Tu R, Wang Q, O’Brart DP (2018). Comparison of standard versus accelerated corneal collagen
cross-linking for keratoconus: a meta-analysis. Invest Ophthalmol Vis Sci.

[25] Wollensak G, Spoerl E, Seiler T. (2003). Riboflavin/ultraviolet-a-induced collagen crosslinking for the
treatment of keratoconus. Am J Ophthalmol.

[26] O’Brart DP, Kwong TQ, Patel P, McDonald RJ, O’Brart NA. (2013). Long-term follow-up of riboflavin/ultraviolet A (370 nm) corneal
collagen cross-linking to halt the progression of
keratoconus. Br J Ophthalmol.

[27] McAnena L, Doyle F, O’Keefe M. (2017). Cross-linking in children with keratoconus: a systematic review
and meta-analysis. Acta Ophthalmol.

[28] Padmanabhan P, Belin MW, Padmanaban V, Sudhir RR. (2021). Extreme corneal flattening following collagen crosslinking for
progressive keratoconus. Eur J Ophthalmol.

[29] Soeters N, van der Valk R, Tahzib NG. (2014). Corneal cross-linking for treatment of progressive keratoconus in
various age groups. J Refract Surg.

